# An Interpretive Description of Patient and Provider Perspectives on the Impact of the COVID-19 Pandemic on Lung Transplant Care Access and Service Delivery in Alberta, Canada

**DOI:** 10.1155/joot/6850873

**Published:** 2025-05-22

**Authors:** Katelyn Brehon, Pam Hung, Maxi Miciak, Rhea Varughese, Kieran Halloran, Kadija Perreault, Paul E. Ronksley, Michael K. Stickland, Jason Weatherald, Douglas P. Gross, Grace Y. Lam

**Affiliations:** ^1^Faculty of Rehabilitation Medicine, University of Alberta, Edmonton T6G 2G4, Alberta, Canada; ^2^Department of Medicine, Division of Pulmonary Medicine, University of Alberta and Alberta Health Services, Edmonton T6G 2G3, Alberta, Canada; ^3^Alberta Respiratory Centre, University of Alberta, Edmonton T6G 2G3, Alberta, Canada; ^4^École des Sciences de la Réadaptation, Université Laval, Québec G1V 0A6, Québec, Canada; ^5^Centre for Interdisciplinary Research in Rehabilitation and Social Integration, Centre Intégré Universitaire de Santé et de Services Sociaux de la Capitale-Nationale, Québec G1M 2S8, Québec, Canada; ^6^Department of Community Health Sciences, Cumming School of Medicine, University of Calgary, Calgary T2N 4Z6, Alberta, Canada; ^7^O'Brien Institute for Public Health, University of Calgary, Calgary T2N 4Z6, Alberta, Canada; ^8^Department of Physical Therapy, University of Alberta, Edmonton T6G 2G4, Alberta, Canada

**Keywords:** access, healthcare quality, interpretive description, lung transplant

## Abstract

**Background:** The COVID-19 pandemic impacted how health services were delivered for patients with chronic pulmonary conditions. To our knowledge, perceptions of patients with lung transplant (LT) and their providers on access to care and service delivery during the COVID-19 pandemic have not been explored in our context of Alberta, Canada. Our objective was to explore LT patient and provider perspectives on the impact of the COVID-19 pandemic on healthcare access and service delivery.

**Methods:** We used interpretive description, a qualitative approach with the end-goal of informing decisions and actions in clinical practice. Interviews were conducted virtually and confidentially transcribed verbatim. Data generation and analysis occurred concurrently. Analysis was informed by Braun and Clarke's six phases of reflexive thematic analysis. Strategies to enhance rigor and trustworthiness of the findings were utilized.

**Results:** We completed 17 interviews: 8 with patients and 9 with providers. Four key themes were generated: (1) “COVID-19 created a relational wall;” (2) “Determining how care should be delivered was a juggling act;” (3) “Balancing supply and demand;” and (4) “The unique costs of being immunocompromised during a global pandemic.” The pandemic impacted social relationships for LT patients, especially through the use of virtual care. Several factors hindered access to care for LT patients. Provider participants highlighted how there were less transplants during the pandemic which created a backlog in transplant surgeries. Fear of COVID-19 meant that some LT patients were hesitant to seek healthcare services, resulting in later-term health consequences. A need for mental health services was identified among this population despite an apparent lack of available services. Participants highlighted the gap in COVID-19 resources that now exists for this population since testing and treatments are no longer as readily available.

**Conclusions:** In conclusion, provider participants did the best that they could with the circumstances they faced to provide high-quality care to LT patients. However, while patient participants were generally understanding of circumstances, LT care suffered as a result of the COVID-19 pandemic. Care for this population generally needs to be in-person, but there is nuance surrounding this recommendation due to LT patients' immunocompromised nature. Health system leaders can leverage our findings to implement learnings from the pandemic and continue to improve services for the ever-growing LT population.

## 1. Introduction

The COVID-19 pandemic impacted how health services were delivered globally. For patients with chronic pulmonary conditions, the pandemic necessitated changes to how care was delivered to ensure COVID-19 safety. Lung transplant (LT) is a strategy utilized when patients with pulmonary conditions and their providers have explored and tried all other possible treatment options; it is a last resort option. To assess how LT programs managed globally during the COVID-19 pandemic, Coiffard et al. surveyed 180 LT centers worldwide [1]. The authors received survey responses from 15 countries and found that 81% of centers reported a decrease in activity and 47% restricted LTs to the most urgent cases [1]. The Canadian Institute for Health Information reported that there was a 17.9% decrease in total adult LTs (i.e., bilateral and single) in Canada in 2020 compared to 2019 [2]. In Alberta, the drastic drop in adult LTs (i.e., bilateral and single) did not seem to occur until 2021 when there were only 53 total adult LTs, representing a 30.3% decrease compared to 2019 (76) and a 19.7% decrease compared to 2020 (66) [2].

Qualitative research has explored the impact of the pandemic on solid organ transplant. A phenomenological study exploring the impact of the pandemic on healthcare access, daily activities, and mental health of patients being assessed or listed for LT during the pandemic found adoption of virtual care led to appointment delays and decreased patient satisfaction with appointments [3]. A descriptive qualitative study aimed at understanding how healthcare providers, administrative assistants, and LT patients were affected by and responded to the COVID-19 pandemic found similar sentiments of displeasure with virtual care due to less physical assessment and decreased ability to read body language cues [4]. However, participants also recognized that virtual care reduced patient cost and burden and was thus a valued care option by patients [4]. Beyond participants' views on virtual care, the pandemic negatively influenced patients' mental health due to isolation, fear of exposure to COVID-19, and general uncertainties about receiving, or living with, a LT during the pandemic [3, 4].

To our knowledge, perceptions of patients with LT and their providers on access to care and service delivery during the COVID-19 pandemic have not been explored in patients who have undergone LT in our context of Alberta, Canada, especially among patients who had a LT prior to the pandemic. Alberta also represents a unique health context as the province has a universal, publicly funded healthcare system that was overseen by a single provincial health authority at the time of the study. The Alberta LT program also provides transplants for northern British Columbia, the Northwest Territories, Saskatchewan, and Manitoba, thus justifying the need to explore experiences in this program during the pandemic as it impacts western Canada rather than a single province. As such, our objective was to explore patient and provider perspectives on the impact of the COVID-19 pandemic on healthcare access and service delivery for patients who had undergone LT prior to the pandemic. While we cannot go back to impact this population's health outcomes during the pandemic, this study will help identify implementable strategies for health system improvements for this group in the postpandemic context and any future pandemic. Learning from this immunocompromised group should also provide insight to manage other immunocompromised populations in the postpandemic context.

## 2. Materials and Methods

This study was conducted in Alberta, Canada (estimated population of 4.8 million in 2024 [5]). Ethics approval was obtained from the University of Alberta's Health Research Ethics Board (Pro00118367) and the University of Calgary's Conjoint Health Research Ethics Board (pSite-22-0025). All participants provided informed verbal consent after reading through the consent form and having an informed consent discussion with the research team.

### 2.1. Researchers' Positionality

The interdisciplinary research team was comprised of clinicians and researchers with diverse levels of experience and various professional backgrounds in pulmonology, public health, rehabilitation, health services, and qualitative and quantitative methodologies. KB, who was the interviewer and primary analyst, has a background in public health and qualitative inquiry, but is not a clinician. Therefore, she relied on balancing the various perspectives from other members of the research team (some of whom provided pulmonary care during the pandemic and others who brought diverse research and clinical experience outside pulmonary care) during data analysis to ensure accurate interpretation and clinical relevance of findings [6].

### 2.2. Methodological Approach

We used interpretive description (ID), a flexible qualitative approach used to address complex questions with the end-goal of creating clinically meaningful, practical findings that inform decisions and actions [6–8]. ID highlights participants' experiences and knowledge to provide in-depth characterization of a phenomenon, therefore aligning with the constructivist paradigm [8]. Findings are a coconstruction of the researchers' inductive interpretations of participant experiences within the context and knowledge of the applied discipline (e.g., medicine, rehabilitation) [6, 8].

### 2.3. Conceptual Framework

Health systems, and individuals' experiences of navigating them, are complex. We used Levesque et al.'s “Conceptual framework of access to health care” to help us construct interview guides that considered how interactions between the health system and patients' abilities to perceive, seek, reach, pay, and engage in healthcare services influenced access [9, 10].

### 2.4. Study Population and Recruitment

In Alberta, LT care is centralized in designated clinics across the province. Rural communities are served by their nearest clinic. All transplants in the province take place in Edmonton, Alberta, regardless of where the patient lives. Before the COVID-19 pandemic, all patients were required to go to Edmonton for a six-week “prehabilitation” period (hereby referred to as prehab) where they would meet their surgeons, care team, and others going through the transplant process as well as learn strategies to improve and/or maintain their physical function while awaiting transplant. Patients that were not from Edmonton were required to remain in the city for 3 months post-transplant before their care was transferred to their local team for ongoing care. The two main clinics in Alberta are located in Edmonton and Calgary, Alberta's two main urban centers.

We aimed to recruit three to five individuals who had undergone LT as well as three to five providers from both the Edmonton and Calgary clinics. All participants were adult residents of Alberta, were willing to participate in research, and were able to provide informed consent. We aimed to recruit individuals who had undergone their LT well before the COVID-19 pandemic rather than those who received a transplant during the pandemic. This recruitment decision meant that patients could not provide thoughts on how the pandemic impacted their pretransplant care. This decision was made since the experience of undergoing transplant during the pandemic could have been an additional factor influencing perceptions of care received. Similarly, provider participants needed to be working in one of Alberta's LT clinics prior to the pandemic. Providers were not required to have a specific minimum number of years of experience. Providers spoke about their perceptions of how COVID-19 impacted patients across the transplant journey from individuals who were pretransplant to those who received their transplants prior to the pandemic. There were no specific inclusion criteria regarding professional designation, and we attempted to gain insight from a variety of professionals including physicians, nurse practitioners, and pre- and post-transplant nurse coordinators.

Purposive sampling toward maximum variation directed patient recruitment. The three primary diversification criteria included age, gender, and geographical location (rural or urban). A diverse sample of individuals who had undergone LT and met inclusion criteria were identified and approached by healthcare providers involved in their care who introduced the study and confirmed consent to share contact information with the research team. The study coordinator (KB) followed up with each eligible and interested individual to provide further information, gain informed consent, and schedule an interview.

All providers working in the included clinics were sent an email by a fellow clinician who was also a member of the research team. The email introduced the study and provided contact information for the study coordinator. Providers willing to participate contacted the study coordinator who provided additional information about the study, gained informed consent, and scheduled an interview. Only one follow-up email was sent to aid recruitment while also minimizing pressure to participate.

### 2.5. Data Generation

Data generation occurred between November 2023 and March 2024. One-on-one semistructured interviews were completed by one interviewer (KB) via videoconference (Zoom Version 6.1.11) or phone. Separate interview guides for individuals who had undergone LT and providers were developed and contained open-ended questions around the topics of need for care, access to care, and quality and effectiveness of care. Probing questions explored topics in greater depth and detail, as needed. Data generation and analysis occurred concurrently, enabling iterative refinements to the interview guides as needed [6].

Participants were asked demographic information including age, and open-ended questions facilitated self-report of gender and cultural or ethnic background. They were also asked the first three digits of their postal code (patients) or years of professional experience (providers). Gender and cultural or ethnic background were open-ended to ensure participants used terminology meaningful to them. These data were collected with the intention of understanding and describing the sample rather than for making comparisons during analysis. Interviews were recorded, transcribed verbatim, reviewed for accuracy, and imported into NVivo Version 14 for analysis [11].

### 2.6. Data Analysis

The analysis was guided by Braun and Clarke's reflexive thematic analysis [12–15]. The primary study analyst (KB) began by familiarizing herself with the data during transcript cleaning (i.e., listening to the audio recording to validate the transcript text) and by rereading all transcripts. Initial analytic thoughts and codes were developed inductively for each transcript. Codes were considered in relation to one another and expanded or collapsed based on patterns of meaning.

A relational approach to analysis was used to generate dialogue between KB, PH, and MM to advance the analysis and reflexively coconstruct findings that were clinically relevant [6]. PH provided a clinical lens by becoming involved at the raw data level through reading each transcript and meeting with KB to discuss important ideas and clinically relevant information as well as to develop iterations of potential themes and subthemes via discussion, critique, and reflection. Themes and subthemes were then named, defined, and exemplary quotes retrieved from the transcripts and shared with MM, who had not read the transcripts. MM provided critical methodological and clinical perspectives on internal coherence within themes and subthemes around a “central organizing concept” [16] and external coherence between themes to ensure they were distinct from one another. Further discussion and critique between KB, PH, and MM occurred until the final themes and subthemes had been generated and defined.

KB contacted each participant to ask whether they would provide feedback on the proposed themes and subthemes [17]. Two provider participants and two patient participants agreed. Proposed themes and subthemes were written in a narrative format that included exemplary quotes. The willing participants were asked to respond to the following questions after reviewing the results document: (1) Do these findings resonate with your experience of LT care during the pandemic?; (2) If an idea does not resonate, why not and what could be changed to make it resonate more?; and (3) Is there anything key to your experience that you think is missing? Any questions or concerns were noted and reflected upon. Subsequent changes were made as necessary. Analysis continued while the final report was drafted and included integration of participant feedback.

### 2.7. Rigor

To enhance trustworthiness, strategies to promote epistemological integrity, representative credibility, analytic logic, and interpretive authority were utilized [6]. We ensured epistemological integrity of the study by choosing ID as our method to address our objective since ID acknowledges the importance of both patient and provider perspectives to understand experiences in the clinical context [6–8]. Interviewing both patients and providers enhanced representative credibility by ensuring both groups contributed to the coconstruction of knowledge. Member-checking with participants as well as researcher discussions also supported representative credibility. Analytic logic was strengthened via the use of verbatim participant accounts, therefore ensuring thick description. Interpretive authority was enhanced through constant reflection during analysis about potential biases or experiences that could impact interpretation.

## 3. Results

### 3.1. Participant Characteristics

We completed 17 interviews: 8 with patients and 9 with providers.  Table 1 (patient) and Table 2 (provider) outline participant characteristics. Most patient participants (62.5%) self-identified as male. Most provider participants (66.7%) self-identified as female. Most patient participants lived in an urban center (87.5%) and had a mean (standard deviation (SD)) age of 59.9 (7.9) years. All providers worked in either the Edmonton or Calgary clinic at the time of the interview and had a range of 4–40 years of experience with a median (interquartile range (IQR)) of 12.0 (10.0–20.0) years.

### 3.2. Impact of the Pandemic on Healthcare Access, Service Delivery, and Health of Individuals With LT

Four key themes were generated related to the impact of the pandemic on healthcare access, service delivery, and subsequent health of individuals who had undergone a LT: (a) COVID-19 created a relational wall; (b) Determining how care should be delivered was a juggling act; (c) Balancing supply and demand; and (d) The unique costs of being immunocompromised during a global pandemic.

#### 3.2.1. COVID-19 Created a Relational Wall

The first theme highlights how the COVID-19 pandemic created challenges to social relationships for individuals who had undergone LT, which are an essential aspect of their ongoing healthcare needs and well-being. There also seemed to be relational impacts perceived by provider participants.

##### 3.2.1.1. Therapeutic Relationships Are Built Through Comprehensive Care and Mutual Trust

Due to the intensive lead up to transplant (i.e., lots of appointments and the prehab program), the 3 months that patients were required to spend with the transplant team in Edmonton following their surgery, and the lifelong, comprehensive care that LT patients receive by the LT care team (i.e., LT team becomes like their “family doctors”), there is an essential therapeutic relationship that is developed between patients and providers on a personal and professional level:

*“They don't really go to their family doctor, their go-to is us. Some of them don't even have a family doctor. They call us their family doctor. And they trust us because we know them so well.”* (Provider 6, female)

Provider participants discussed how these relationships made it easier to gauge which of their longer-term patients (i.e., had transplant a while ago) were able to reliably and accurately self-monitor their condition during the pandemic. Patient participants also seemed to reciprocate this trust in their providers who became their go-to sources of information and answers to any questions/concerns during the pandemic:

*“it feels like we're all family. … They care enough where they'll really go out on a limb for you. … I can walk in to absolutely any appointment and be treated so well that it's disgusting in how wonderful everybody is. … it's just so nice to have that camaraderie, that relationship, as opposed to just going in and you're a number. And if you're really full of fear and a great deal of apprehension about walking into a situation where it's life or death, then to have that given to you, it just makes everything that much better.”* (Patient 5, male)

##### 3.2.1.2. Hindered Ability to Access Supportive Relationships During the COVID-19 Pandemic

For newer transplant patients (i.e., awaiting transplant or recently transplanted), provider participants discussed how it was more challenging to develop therapeutic relationships using virtual care. Provider participants also discussed how virtual care negatively impacted the ability of newer patients and their families to develop relationships with other transplant patients and their families in the prehab program. Relationships between patients going through transplant at similar times and between families whose loved ones are going through transplant are essential from a social support perspective. Social support was also limited during the pandemic within the hospital and clinic settings as there were varying restrictions as to whether family members and/or friends could be in these facilities with patients. Support people play a major role in transplant care (i.e., information retention, communication, care of loved ones) so these limitations impacted patient care. Finally, due to increased vulnerability of this population from being immunocompromised, patient participants discussed sometimes isolating themselves from social circles because they were afraid of getting COVID-19. Coupling this social isolation with the fact that patients did not get to see their care team as often during in-person appointments meant that this population was more socially isolated during the pandemic than ever before.

*“I don't mean to inflate our importance in these patients' lives,… I feel at this time the patient feels so isolated that clinic appointments are kind of their form of socialisation for some… And so not having that sense of community definitely … impacted them”* (Provider 5, female)

*“I kind of missed seeing people. Because you know, you build relationships over the years and so it becomes part of your routine every month or every two or three months, you meet up with these people … and you talk about this and that for a bit.”* (Patient 1, male)

#### 3.2.2. Determining How Care Should Be Delivered Was a Juggling Act

The second theme discusses the various factors being considered and balanced when determining how LT patients would be accessing care during the pandemic.

##### 3.2.2.1. Needing, Accessing, and Providing In-Person Care in a Strained System

Regardless of whether there was a pandemic happening or not, there were some LT patients that needed to be brought into the clinic or hospital for care because they were experiencing an infection or exacerbation. Patients could always be seen in-person if they needed to be: *“If I needed it,… I would have gotten it. Whatever I needed, I would have access to”* (Patient 5, male). However, bringing people in for in-person outpatient care became a balancing act between needing certain tests and having limited access to testing facilities or the required healthcare personnel to conduct testing. If tests could not be arranged on an outpatient basis or if the patient required more comprehensive care, a balance was sought between bringing people to the hospital, hospital capacity (which was challenged during the pandemic), and potential infection risks to the patient. Provider participants discussed how intensive care capacity was challenging during the pandemic and how sometimes people that should have been admitted were not admitted or admitted later than they should have been. However, patient participants did not perceive that they could not get the care that they needed when they needed it. If patient participants were admitted to hospital, they seemed to feel like they received quality care.

*“Yeah, so [access] went down … in terms of what programs we're offering and how many physicians were working based on needing to do other streams of work that the pandemic brought about. Also,… because of COVID, like people just get sick … there was kind of just a general decrease in access and increase in wait times … if anything absolutely needed to get done we just sent our patients to hospital so that we can still … get it done. But it's just the convenient, outpatient timing … was more challenging.”* (Provider 5, female)

##### 3.2.2.2. COVID-19 Safety Measures Impacted Perceived Safety of Care

LT patients who were viewed as stable were often triaged to virtual care and phone assessments:

*“For a long time there… was nothing in person. And … I didn't really have any issues either. I didn't have any issues at all during that period. So there was no real reason for me to go in. If there was a reason to go in, then they would do it.”* (Patient 4, male)

This triaging to virtual care was done to minimize COVID-19 infection risk but came at the cost of perceived (by providers) lower-quality assessments which may have meant care safety or quality was impacted if proper assessments could not be completed. While self-monitoring tools were in play, as mentioned above, some patients were less reliable at doing self-monitoring and/or reporting their results from using such devices. Those that were viewed as less reliable with self-monitoring were often triaged to in-person care for their regular appointments to prevent any missed exacerbations or infections. Patients now seem somewhat more reluctant to come to clinic because they have enjoyed the convenience of virtual care:

*“I think … the fallout … is that the pandemic went for so long that people have become comfortable to that method of follow-up, that they don't want to come to clinic anymore … I understand that because there are a lot of patient benefits. But at the same time, we don't get the formalized testing that we want. And there's a lot more resistance to come into clinic … because ‘hey, well, we did it for three years and I'm still alive, so let's continue to do that'”* (Provider 9, male)

##### 3.2.2.3. The Trade-Offs of Increased Prehab Accessibility

During the pandemic, access to the prehab program became easier at certain points when it was shortened from 6 weeks to 4 weeks (sometimes only 2 weeks) and adapted from in-person to virtual delivery:

*“Having to relocate to another city for three months post-transplant and for that [prehab] program, is clearly an access issue. We do a pretty good job, I think, at helping support them financially and with those things, but again, they have to relocate to another city away from their home, their lives, to be able to manage that.”* (Provider 8, female)

However, while virtual prehab was more cost-effective and accessible as patients from out of town did not have to relocate to Edmonton while also paying living expenses where they were from, it meant that they did not establish the same social connections with their care team and other patients and families, which is viewed as an important aspect of the program. Additionally, virtual prehab meant that providers experienced challenges providing education about exercise techniques and could not assess how patients were doing the prehab activities, meaning that some patients may not have been getting the full benefit of the prehab program before having their transplant:

*“I think another big impact … is the education component that they would have missed. … families now say how important it was to connect with others that were kind of going through the same thing. And you miss all that. And then the same with the education piece. … I think that lived experience and being within that prehab program and hearing other people's questions,… all of that is gone [with virtual].”* (Provider 7, female)

#### 3.2.3. Balancing Supply and Demand

The third theme highlights how the pandemic impacted certain factors related to supply and demand of transplant services, thus impacting patient care.

##### 3.2.3.1. The Transplant Log Jam

Provider participants discussed how there were less LT surgeries during the pandemic. Initially, this decrease was due to uncertainty about whether the lungs from someone who had previously had COVID-19 were transplantable coupled with a general lack of available organs to transplant because traumas that would contribute to organ donation were not occurring at the same rate as prior to the pandemic: *“initially in the pandemic, people just weren't going out as much. There was less trauma, there were less donors around the country. And so we saw transplant numbers fall just because there were less donors”* (Provider 2, male). Limited intensive care unit space to support the care needed after the transplant surgery was also a factor preventing LTs: *“But even our numbers of post-transplant went down significantly because there just wasn't capacity in the ICUs”* (Provider 9, male). Due to the limited number of available organs and some patients getting sick with COVID-19 while awaiting transplant and needing to be taken off the list temporarily, some people died while waiting for a transplant or had to be relisted after their COVID-19 infection resolved thus delaying their surgery.

##### 3.2.3.2. Steady Stream Now a Raging River

Before COVID-19, the LT program in Alberta was already busy and struggling to meet demand, especially with developments in broadening the eligibility criteria for transplant:

*“Lung transplantation has been a growing field for decades and continues to grow exponentially. … we've expanded the criteria of who could be eligible for a transplant and when you do that, then of course your potential number of recipients very quickly increases.”* (Provider 2, male)

The clinic has been working to hire more doctors, which had mixed reviews from patient participants. Some did not like the increased number of doctors and being seen by new providers or not seeing the same provider each appointment: *“they're bringing in some new [doctors] and I don't feel they know us as well and some things fall through the cracks there”* (Patient 2, female). Other patient participants thought different doctors brought different perspectives to their care:

After learning how to live with COVID-19 and as health services opened up again, the busyness of the clinic became more challenging because there was still some limited access to testing and appointments. There was also an influx of patients waiting to be seen in-person finally (i.e., existing patients and new referrals). Due to the disruptions and changes in the normal transplant program during the pandemic, provider participants found they were performing transplants on higher-risk patients when COVID-19 restrictions were lightened:

*“We found a big log jam … when COVID started to lighten up. There were so many very, very sick people on the list and … they'd come to us in worse shape and it would take longer to recover. But also put them at risk for all kinds of complications like infection. … they're much more deconditioned coming into us, so then they recover so much slower … it was a cascade of events for sure.”* (Provider 3, female)

##### 3.2.3.3. Needing a Mental Health Life Raft for Support

From patient and provider participant perspectives, mental health is a major factor associated with LT. Mental health access for patients in Edmonton seemed to always be challenging. Access was better in Calgary because of access to transplant psychiatrists; however, providers have always advocated for access to psychological counselling to complement psychiatry. During the pandemic, one psychiatrist left the transplant program, decreasing access for patients. Further, because of increased need for mental health services in the general population, access to other mental health resources became even more challenging for patients to access. Supply definitely did not (prior to the pandemic) and currently does not match demand in this population.

#### 3.2.4. The Unique Costs of Being Immunocompromised During a Global Pandemic

The final theme discusses unique challenges that individuals with LTs and their providers faced when navigating being immunocompromised or working with immunocompromised patients, respectively, during the COVID-19 pandemic.

##### 3.2.4.1. The Consequences of the Fear of Being Vulnerable

Fear of contracting COVID-19 stopped some LT patient participants from seeking health services during the pandemic:

*“Fear of needing to go to the hospital because … at the beginning of the pandemic all we knew was that there was this new virus and that people were dying and that we were incredibly vulnerable because we're immunocompromised. More vulnerable than other people.”* (Patient 7, female)

However, instead of having an entirely protective effect (i.e., protecting them against contracting COVID-19), this meant that some transplant patients experienced costs related to their health later on. Provider participants discussed how some patients' cancers went undetected until later stages or that their lung disease had progressed extensively by the time that they went in person to seek care, for example:

*“I would dare say that cancer is actually something that I would be able to say confidently … would've been caught sooner [if there was no pandemic] … incidence of cancers are just so much higher in our patient population because of the immune suppression. I can say that … some screening was definitely put off or missed for entire years within that timeframe.”* (Provider 8, female)

##### 3.2.4.2. Complications of Testing Positive for Months

During the pandemic, it was recognized by participants that transplant patients often test positive for COVID-19 for a long time after initial infection because their bodies cannot clear the virus as effectively as others due to the immunosuppressants they are taking:

*“We've had people who had COVID for like three months or four months and it just took forever to clear it. And it's really hard to know what to do in those situations. So there are some people who really suffered because they were limited from being exposed to rehab and things like that, because they were still shedding for three months. They kind of decompensated, deteriorated, and we've had people die because they just weren't able to get out of the hospital.”* (Provider 9, male)

*“I had COVID for eight months. And I was in and out of the hospital for most of that. And it was pretty awful. … They tried everything they could think of. Every week they'd test me for COVID and it would come back as positive. … it was a very stressful time”* (Patient 3, female)

These long-term periods of patients testing positive for COVID-19 came at the cost of provider participants needing to learn how to manage these patients' healthcare as many of them still needed to be seen in-person. However, bringing these patients in for in-person care was risky in a clinic with other immunocompromised patients.

##### 3.2.4.3. When the World Moves on Without You

When the pandemic was seemingly over for the general population, the LT population still needed access to resources like COVID-19 tests and therapies because of their immunocompromised nature. Patient participants discussed how outpatient resources were challenging to access and only got more challenging as pandemic restrictions relaxed for the general population:

*“… just the lack of knowledge and … knowing where do you go for a test? Because we had to obtain a COVID test. They didn't have home tests at the beginning, and … to get the antivirals you still had to get a formal test, and that process was absolutely horrific. And they did not isolate the immunocompromised from everyone else who needed a test. So you were often … in a line-up with people who weren't masking, who were coughing and sick. And that was terrifying and horrible.*” (Patient 7, female)

Provider participants discussed having to find workarounds to provide some of the services in clinic so that LT patients had access (i.e., they still need a positive COVID-19 test to access COVID-19 treatments even though testing is no longer widely available):

*“I would say that we're now in a place again where we don't have resources in an outpatient world because COVID still very much exists in this clinic, and it affects these patients very detrimentally, but there's no outpatient resources for swabbing, etc., anymore. So they … have to come here to clinic for us to do it,… but then we're bringing somebody that's potentially infectious into an immune-compromised space.”* (Provider 8, female)

## 4. Discussion

We explored patient and provider perspectives of the impact of the COVID-19 pandemic on LT healthcare access and service delivery. The pandemic and use of virtual care seemed to impact LT patients' relationships with providers, other patients and their families, and their social circles. While there was limited access to outpatient testing and bringing patients into hospital was challenging at times due to hospital capacity, patients felt that they could always access in-person care if it was necessary. Stable patients who had received their transplant well before the pandemic tended to be triaged to virtual care compared to patients who received a transplant more recently. Virtual care seemed to sometimes come at the cost of less comprehensive assessments but was valued because of its convenience and COVID-19 safety. Perceptions of virtual prehab were mixed given that it was more affordable and accessible for patients but came at the cost of patients being unable to establish social connections with providers and peers, which are essential in LT care. Provider participants highlighted how there were less transplants during the pandemic due to limited intensive care unit space, decreased organ availability, and COVID-19 uncertainties. This created a backlog in transplant surgeries which, when coupled with broadening LT criteria, has resulted in Alberta's transplant center now struggling to meet demand. There is also higher demand for mental health services among this population but there is an apparent lack of available services. Fear of COVID-19 meant that some LT patients were hesitant to seek healthcare services during the pandemic, resulting in cancers diagnosed later or at a more progressed disease state. Learning how to manage LT patients who had been testing positive for COVID-19 for months due to their immunocompromised nature created challenges for providers. Finally, participants highlighted the gaps in COVID-19 resources that now exists for this population since the rest of the world has moved on from the pandemic.

Our participants discussed how the pandemic impacted relationships between patients and providers, other patients and their families, and their wider social circles. In a phenomenological study (*n* = 22) exploring the potential impact of the pandemic on access to healthcare, daily activities, and coping and stress in patients who were being assessed or listed for LT, Mansell and colleagues found that most patients experienced decreased social activity which resulted in negative impacts on their mental well-being [3]. Similarly, in a convergent parallel mixed-methods study focused on illuminating how the pandemic impacted transplant journeys in Canada, Fox et al. described participants taking extra precautions to stay safe against COVID-19 at the cost to their social health [18]. These sacrifices extended outside of normal day-to-day activities and often existed in healthcare facilities, with support people unable to visit patients due to pandemic restrictions [18]. Taken together, these findings show that there was a social cost of the pandemic for LT patients and their circles.

To promote COVID-19 safety, virtual care was widely adopted within healthcare with the transplant space being no exception. Our participants described mixed perceptions of virtual care. Virtual assessments were perceived as less comprehensive since providers could not fully observe patients. However the convenience, safety in terms of COVID-19, and cost-effectiveness for patients of virtual care were valued. Similar benefits of virtual care within the transplant space have been described in the literature [18]. Fox and colleagues found that virtual care significantly reduced travel time and daily life disruption [18]. Similarly, in a descriptive qualitative study investigating how hospital stakeholders were impacted by and responded to the pandemic (*n* = 16), Puerto Nino et al. found virtual care resulted in a significant reduction in patient expenses and travel time and was thus strongly favored by patients [4]. Disadvantages related to delayed care and challenges with scheduling and accessing physicians [3] as well as the lack of physical examination [4] have also been described. This highlights the need for a combination of both virtual and in-person modalities to be included in regular LT care in the postpandemic context and during any future pandemics that may occur, if possible. This recommendation extends to the prehab program as well. In our study, the prehab program experienced several changes in length and mode of delivery in response to the changing pandemic climate. To balance flexibility and lessening patient expenses (i.e., related to time, travelling, relocation) with the essential psychosocial and physical functioning benefits of the prehab program, future research should investigate the effectiveness of a mixed virtual and in-person prehab program among this population.

Provider participants in our study highlighted how there were less transplants during the pandemic due to limited intensive care space, decreased organ availability, and general uncertainties around COVID-19 which created a backlog in transplant surgeries. Alberta's transplant center is now struggling to meet demand. Alberta does not seem to be unique in terms of decreased transplant numbers during the pandemic with several authors [4, 19–21] describing fewer transplants during the pandemic than before. The most commonly described reasons for less LTs during the pandemic were lockdowns [19], reduced critical care capacity [4, 19–21], decreased resource availability [4], and reduction in organ donation and procurement [4, 21]. Given that severe COVID-19 infection created a new reason for LT, it is troubling that clinics in Alberta are struggling to meet demand. In the postpandemic context, LT clinics should aim to hire more clinical personnel to help manage the backlog of patients. Continued efforts are needed to highlight the importance and lifesaving nature of organ donation, thus hopefully resulting in more people registering as organ donors.

Participants discussed the mental health impacts of LT that got more challenging during the pandemic and the lack of mental health services to properly support this population. Mansell and colleagues highlighted how mental health challenges for patients going through or awaiting transplant are high since they are coping with facing their own mortality and living with an advanced disease that can be associated with loss of independence and social roles [3]. Layering a global pandemic on top of this created additional stressors such as social isolation and disrupted work and financial situations [3]. Our participants discussed how even before the pandemic, there were regional disparities how mental health challenges were managed. Fox et al. support this finding with both of their participant groups discussing how LT patients' mental health was not adequately supported [4, 18]. In the postpandemic context, more resources are needed to address the mental health impacts of the pandemic for immunocompromised individuals, like LT patients. Mental health resources should extend beyond psychiatric support with more availability of psychologists trained in working with immunocompromised populations. These additional resources may help manage the additional mental health burden that resulted from the COVID-19 pandemic and may also help manage these challenges proactively during any future pandemic.

Learning how to navigate a world with COVID-19 was challenging for our participants. Provider participants had to learn how to manage LT patients testing positive for COVID-19 for months on end while still needing access to regular care. The literature suggests that an infectious disease physician has an important role to play in navigating such cases [22], which are present in the basket of services provided to LT patients in Alberta. Learning how to manage a highly vulnerable population during a global pandemic was essential while it was happening as there were no other options. However, these learnings can also be applied to any future pandemic contexts and may also simply apply to managing immunocompromised states during periods of higher illness in the general population, such as cold and flu season. Both patient and provider participants in our study had to learn how to access COVID-19 resources and treatments (i.e., COVID-19 testing, therapies, ongoing access to vaccines) after they stopped being widely available to the general public. This proved challenging for some of the patient participants and highlights the need for ongoing support and advocacy from the LT healthcare team [18] in the postpandemic context and during any future pandemic that may occur.

### 4.1. Limitations

Some limitations may impact the transferability of our findings. First, we interviewed LT patients who received their LT well before the pandemic instead of during the pandemic. This decision was intentional as one of the goals of the study was to draw comparisons between care delivery prior to and during the pandemic. However, we were unable to provide the patient perspective of those who underwent LT during the pandemic. Future research should explore the impact COVID-19 had on those who received their transplants during the pandemic to understand how their experiences may have varied from those who received their transplants before COVID-19. Second, we interviewed mainly patient participants from urban centers and mainly female provider participants. While we likely could have improved the diversity of patient participants with further recruitment since the two clinics we recruited from serve everyone in the province, clinic appointment frequency and feasibility limited availability to do so. Diversity of provider participants was constrained as there is a limited number of providers working in the clinics, so the population to recruit from was small. Future research should include more gender and geographically diverse samples to strengthen transferability and understand if perceptions of access and service delivery differ from the current study's findings.

## 5. Conclusions

We studied the impact of the pandemic on LT healthcare access and delivery by integrating patient and provider perspectives to identify implementable strategies for postpandemic health system improvements. In conclusion, provider participants did the best that they could with the circumstances they faced to provide high-quality care to LT patients. However, while patient participants were generally understanding of the circumstances, LT care suffered as a result of the COVID-19 pandemic. The pandemic negatively impacted social relationships for LT patients. Several factors hindered access to care for LT patients; however, they felt they could always access in-person care if it was necessary. Provider participants highlighted how there were less transplants during the pandemic which created a backlog in transplant surgeries in Alberta. Since the Alberta program also provides transplants for northern British Columbia, the Northwest Territories, Saskatchewan, and Manitoba, the pandemic-related decline in transplant surgeries affected all of western Canada as well. Fear of COVID-19 meant that some LT patients were hesitant to seek healthcare services, resulting in later-term consequences to health such as undiagnosed cancers or more progressed disease. There is also high demand for mental health services among this population but there is an apparent lack of available services. This lack of mental health services is a crisis Alberta-wide and is not unique to the transplant program representing a critical area for future investment and resourcing. Finally, participants highlighted the gap in COVID-19 resources that now exists for this population since the rest of the world has moved on from the pandemic.

Health system leaders can leverage our findings to implement learnings from the pandemic and continue to improve services for the ever-growing LT population. Our findings may also be relevant for rapidly adapting service provision in future pandemic climates, should the need arise.

## Figures and Tables

**Table 1:** Patient participant characteristics (*n* = 8).

Variable	*n* (%) or mean (SD)
Gender^a^	
Male	5 (62.5%)
Female	3 (37.5%)
Mean age (years) (SD)	59.9 (7.9)
Rural/urban	
Rural	1 (12.5%)
Urban	7 (87.5%)
Ethnicity^b^	
Caribbean	1 (12.5%)
Caucasian	5 (62.5%)
Ukrainian	1 (12.5%)
Italian	1 (12.5%)

^a^All participants were asked their gender as an open-ended question in conversation with the interviewer. All responded using sex-based categories of male/female. We are reporting terms that participants used.

^b^All participants were asked to identify their cultural or ethnic background in an open-ended question in conversation with the interviewer. We are reporting terms that the participants used.

**Table 2:** Provider participant characteristics (*n* = 9).

Variable	*n* (%), median (IQR), or mean (standard deviation (SD))
Type of provider	
Physician	5 (55.6%)
Surgeon	0 (0.0%)
Allied health (i.e. registered nurse, nurse practitioner)	4 (44.4%)
Gender^a^	
Male	3 (33.3%)
Female	6 (66.7%)
Mean age (years) (SD)	43.3 (6.6)
Median years of experience (IQR)	12.0 (10.0–20.0)
Ethnicity^b^	
Asian	6 (66.6%)
Caucasian	3 (33.3%)

^a^All participants were asked their gender as an open-ended question in conversation with the interviewer. All responded using sex-based categories of male/female. We are reporting terms that participants used.

^b^All participants were asked to identify their cultural or ethnic background in an open-ended question in conversation with the interviewer. We are reporting terms that the participants used.

## Data Availability

The dataset generated and analyzed during the current study is not publicly available as we did not get consent from participants to share their data publicly. However, the dataset can be made available from the corresponding author on reasonable request.
